# Pneumatic Bur Engine

**Published:** 1870-04

**Authors:** 


### PNEUMATIC BUR ENGINE.

We have had in use one of these instruments for about two months, and can not but express our entire satisfaction with its working, especially so far as the operation of the finishing bur is concerned. It is a great labor saving appliance in the work of finishing all fillings where the bur can be brought to bear; it also reduces the time of finishing to less than one-half that required by the ordinary mode, besides doing the work better. It also operates the excavating bur and drill very efficiently and rapidly. The construction is such that the burs and drills may be run at any angle with the main shaft that may be desired, so that posterior surfaces may be reached quite as well as anterior when the contiguous teeth will permit. There is also an attachment for using the file and the plugging instrument; with these we have no experience, and are not prepared to speak of them, but so far as we have become familiar with the instrument, we regard it an invaluable one, and should be in the hands of every good operator. Dr. E. R. E. Carpenter, of Chicago, will furnish them, or give any information that may be desired. T.

